# Foreign Body Aspiration of a Dental Bridge in the Left Main Stem Bronchus

**DOI:** 10.1155/2012/798163

**Published:** 2012-10-10

**Authors:** Monay Mahmoud, Syed Imam, Hetalben Patel, Matthew King

**Affiliations:** Department of Internal Medicine, Meharry Medical College, Nashville, TN 37208, USA

## Abstract

Aspiration of tracheobronchial foreign bodies is a life-threatening event that occurs mainly in children. Occurrence in adults is rare and usually has a subtle presentation as most adults are unaware of aspiration of any foreign material. Decreased levels of consciousness, sedation, and neuromuscular diseases are major risk factors for foreign body aspiration in adults. Prompt diagnosis and intervention through foreign body retrieval are critical to prevent significant morbidity and mortality. Retrieval procedure is risky, and sudden decompensation of the patient can occur anytime. We are presenting an adult who accidentally aspirated his dental prosthesis during sleep and underwent successful retrieval of the dental bridge using flexible bronchoscopy.

## 1. Introduction

Foreign body aspiration is an uncommon problem in adults [1]. About 80 percent of reported cases occur in children under 15 years of age [2]. Foreign body aspiration can be an emergent life-threatening condition if the aspirated object is large enough to cause complete airway obstruction. In the United States, approximately 500–2000 deaths occur each year from foreign body aspiration [3]. Here, we report a case of a 48-year-old male who aspirated his dental prosthesis during sleep and underwent successful retrieval of a dental bridge from the left main-stem bronchus using a flexible bronchoscopy.

## 2. Case Presentation

**Figure 1:**
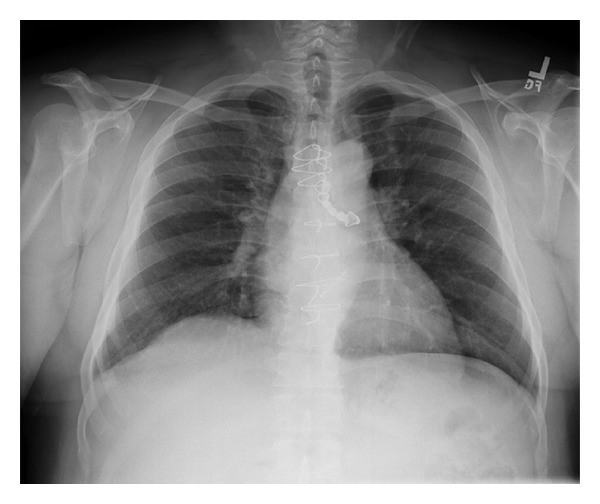
Chest radiograph shows a dental prosthesis lodged in the left mainstem bronchus.

**Figure 2:**
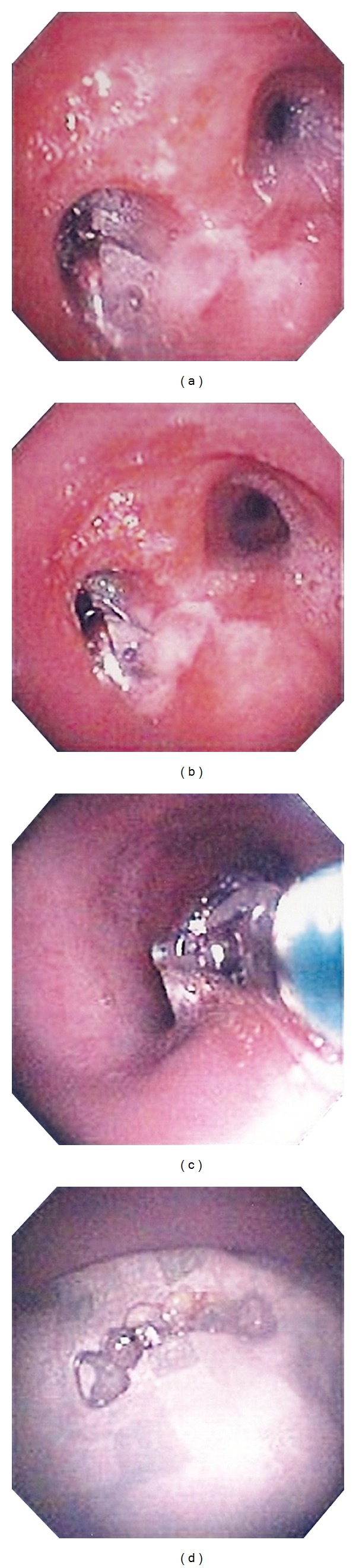
Bronchoscopic images obtained during extraction of the dental prosthesis. (a) and (b) depict the prosthesis within the left main stem bronchus. (c) demonstrates the endobronchial forceps grasping the prosthesis during extraction as it is passing through the mid trachea. (d) shows the prosthesis on a bedside tray after removal.

A 48-year-old Hispanic male with a past medical history of asthma and aortic regurgitation status post aortic valve replacement presented to the emergency room due to acute onset of dyspnea. The patient awoke feeling very short of breath. He experienced some cough productive of white sputum as well as general respiratory discomfort without any fever, hemoptysis, or chest pain. Upon arrival to the emergency department, he was saturating 95 percent on room air and all vital signs were within normal range. Physical exam was significant for decreased air entry on the left side. The remainder of the physical exam was unremarkable. Chest X-ray revealed a dental prosthesis in the left main stem bronchus (Figure 1). The patient denied the use of any sedative medications or alcohol intake and reported that it must have occurred during sleep. He was admitted to the intensive care unit and underwent flexible bronchoscopy. After administration of 2 mg of versed and 100 mcg of fentanyl, a bronchoscope was introduced orally. Topical lidocaine was administered to the glottis and the trachea was entered. A dental prosthesis was identified in the left main-stem bronchus just past the carina (Figure 2). Endobronchial forceps were used to grasp the wire frame of the dental prosthesis. Light traction was applied and the dental prosthesis was slowly dislodged. With the prosthesis grasped by the forceps, the bronchoscope, forceps, and prosthesis were extracted en bloc through the vocal cords. The patient was observed for a few hours during which he reported dramatic improvement of dyspnea and he was discharged in a stable condition. The dental prosthesis was returned to patient with instructions to remove it prior to sleep until more permanent fixation could be achieved.

## 3. Discussion

Airway foreign bodies are a major cause of morbidity and mortality in the United States. Foreign body aspiration is sometimes referred to as a café coronary (elderly adults) [4]. Foreign bodies have a tendency to lodge in the right main stem bronchus as it is more vertical and larger in diameter than the left main stem bronchus. However, as in this case report, foreign bodies may enter the left main stem bronchus and, in fact, have been reported in all airway locations. The nature of aspirated foreign bodies varies significantly with the geographical and cultural differences throughout the world. Food is the most common aspirated foreign body. Nuts, seeds, pins, nails, and dental appliances following dental procedures have all been documented [5, 6]. Dental prosthetics such as the dental bridge aspirated by our patient represent up to twenty-seven percent of cases [7, 8].

Since foreign bodies are typically stuck distally in the lower lobe bronchi or the bronchus intermedius, acute presentation in adults is rare; however, life-threatening asphyxia and sudden decompensation secondary to complete obstruction may occur [9]. Our patient presented with cough which is the most common presenting symptom. Other symptoms include fever, chest pain, and hemoptysis. Dyspnea was reported by our patient but is generally a rare presenting symptom. Physical examination of adults with foreign body aspiration is often unrevealing. Stridor, wheezing, or diminished breath sounds may be encountered if the degree of obstruction is severe enough.

Foreign bodies can remain undetected for months in adults which require a high index of suspicion for diagnosis as most adults do not recall a history of choking [10]. Many foreign bodies are incidentally seen on radiographic imaging ordered for symptoms mistakenly attributed to other medical conditions including asthma and unresolving recurrent pneumonia [10, 11]. If a diagnosis of foreign body aspiration is delayed, a retained foreign body may result in unresolving pneumonia, lung abscess, and bronchiectasis. Also, formation of granulation tissue around the foreign body may occur and may resemble bronchogenic carcinoma [12].

Foreign body aspiration is a well-recognized potential complication in orthodontic practice [7]. Numerous risk factors may facilitate foreign body aspiration in adults including impairment of swallow reflex and ineffective airway protective mechanisms with aging, alcohol, or sedative use, altered sensorium, neurological dysfunction, and loss of consciousness. Several preventive orthodontic techniques are employed to decrease the occurrence of foreign body aspiration with both removable and fixed appliances. Adherence to appropriate technique in placement of dental prosthesis, following standard operating procedure, regular inspection of appliances, and timely replacement is of paramount importance in preventing events similar to our case [7].

Therapeutic removal of foreign bodies is not a new concept. The earliest report of airway foreign body removal was performed via bronchotomy by Louis in 1759 [13]. The first endoscopic removal of a foreign body occurred in 1897 [14] and since then bronchoscopy has remained the gold standard for evaluation of patients with high clinical suspicion for foreign body aspiration.

Our case describes the successful extraction of a large foreign body using fiber optic bronchoscopy. The success rate of fiber optic bronchoscopic extraction in adults ranges from 60 to 90 percent [3, 15]. The procedure is usually done under local anesthesia. Despite a high success rate, fiber optic bronchoscopy extraction entails some risks. The fiber optic forceps provide less grasping power of a foreign body compared to rigid bronchoscopy forceps which may result in migration of the foreign body to the contralateral lung and can lead to a fatal outcome [16]. For this reason, one should have a low threshold for conversion to a rigid bronchoscopy.

Fiber optic is a preferable option in contrast to rigid bronchoscopy in the case of a distally wedged FB, in mechanically ventilated patients, or in the presence of spine, craniofacial, or skull fractures that prevent the manipulation required for rigid bronchoscopy [3]. In younger children, the optimal extraction technique is an outstanding issue currently under debate. Surgical extraction of foreign body through bronchotomy or even segmental resection under general anesthesia is the last resort if extraction using bronchoscopy is unsuccessful. Presence of an experienced thoracic surgeon and anesthesiologist is essential to prevent significant morbidity and mortality associated with unsuccessful attempts of retrieval using bronchoscopy. 

The use of corticosteroids and antibiotics in the setting of foreign body aspiration is controversial. It is usually recommended to use a short course of corticosteroids before FB removal when a well-tolerated FB is encased in a bulky and bleeding granulation tissue [17]. Corticosteroids are not recommended as a prophylactic measure for postoperative subglottic edema; however, it can be used in conjunction with aerosolized epinephrine or helium oxygen therapy in cases of established subglottic edema. Antibiotics are not indicated except in the setting of documented respiratory tract infection.

Immediate removal of tracheobronchial foreign body is essential to prevent life-threatening complications. Bronchoscopic extraction of foreign body even by a skilled operator is not a risk-free procedure and it has to be done in a monitored setting with complete resuscitative measures available as acute decompensation may occur secondary to accidental dislodgment of the foreign body. Procedure outcome varies significantly by the type and location of the obstructing foreign body as well as the level of experience of the bronchoscopist.

Fortunately for our patient, the wire frame of the dental prosthesis was firm and narrow, providing an excellent grasping point for the endobronchial forceps that facilitated extraction of the dental prosthetic. At his outpatient follow-up visit, the patient was doing well, with complete resolution of dyspnea. He is still using his dental prosthetic, but not during sleep.
